# Supplementary material for: Daily Rhythms in the IGF-1 System in the Liver of Goldfish and Their Synchronization to Light/Dark Cycle and Feeding Time
Source: Animals (Basel). 2022 Nov 30;12(23):3371. doi: 10.3390/ani12233371 (PMC9739714; doi:10.3390/ani12233371)
Supplement: Supplementary file 1 [file animals-12-03371-s001.zip › animals-2057123-supplementary.pdf]

Gene	Access number (GenBank)		Primer sequences (5′→3′)	Product (bp)
ef-1α	AB056104.1	**F**	CCCTGGCCACAGAGATTTCA	101
		**R**	CAGCCTCGAACTCACCAACA	
igf-1	AF001006.1	**F**	CAGGGGCATTGGTGTGA	153
		**R**	GCAGCGTGTCTACAAGC	
igf1ra	AF216773.2	**F**	GCGTGTAACCACGAGATCCA	132
		**R**	GCACTGTTCCTTCTCCCCTC	
igf1rb	AF216772.2	**F**	CCCTTCACCGTTTACCGCAT	155
		**R**	GAACACAGAACCCTCCAGATGT	
igfbp1a-a	XP_026089341.1	**F**	ACAACACGCCAGATCACTCC	183
		**R**	TATGACAAGGACCCTGCTCC	
igfbp1a-b	XP_026147113.1	**F**	AACACGCCAGATCAGCCG	184
		**R**	TCAATATGACAAGGACCCTGTTGT	
igfbp1b-a	XP_026052969.1	**F**	ACGCCGTCTGTGTAGAGTTT	217
		**R**	ATGACGTCCAGAGCTGCGT	
igfbp1b-b	XP_026062900.1	**F**	CCGTCTGCACCGAGGAC	218
		**R**	AGTGATCAGATTCAGAGACGCTC	
igfbp2a-a	XP_026070558.1	**F**	TGGTGAATGCTGGTGTGTGG	113
		**R**	AGGGATCCATCTCTAGGGCA	
igfbp2a-b	XP_026118614.1	**F**	GAGGACCCTAAAGCACCGC	237
		**R**	GGGTCTTCCTGTGTGTGGATTA	
igfbp2b-a	XP_026073308.1	**F**	GCGTGTCCCAAGCTCACC	159
		**R**	CGGTAACTCTGAATTGGCGCTT	
igfbp2b-b	XP_026127022.1	**F**	GGCGTGTCCCAAGCTCATA	160
		**R**	CTGTAACTCTGAATTCGCGGTC	

F: Forward, **R**: Reverse
